# A Novel Triculture System (CC3) for Simultaneous Enzyme Production and Hydrolysis of Common Grasses through Submerged Fermentation

**DOI:** 10.3389/fmicb.2016.00447

**Published:** 2016-03-31

**Authors:** Vincent V. Leo, Ajit K. Passari, J. Beslin Joshi, Vineet K. Mishra, Sivakumar Uthandi, N. Ramesh, Vijai K. Gupta, Ratul Saikia, Vijay C. Sonawane, Bhim P. Singh

**Affiliations:** ^1^Molecular Microbiology and Systematics Laboratory, Department of Biotechnology, Mizoram UniversityAizawl, India; ^2^Department of Biotechnology, J.J College for Arts and SciencePudukkottai, India; ^3^Biocatalysts Lab, Department of Agricultural Microbiology, Tamil Nadu Agricultural UniversityCoimbatore, India; ^4^Molecular Glyco-biotechnology Group, Department of Biochemistry, National University of Ireland GalwayGalway, Ireland; ^5^Biotechnology Division, CSIR-North East Institute of Science and TechnologyJorhat, Assam, India; ^6^Biochemical Engineering Research and Process Development Centre (BERPDC), Institute of Microbial TechnologyChandigarh, India.

**Keywords:** perennial grasses, lignocellulosic biomass, hydrolytic enzymes, FT-IR, triculture, SEM

## Abstract

The perennial grasses are considered as a rich source of lignocellulosic biomass, making it a second generation alternative energy source and can diminish the use of fossil fuels. In this work, four perennial grasses *Saccharum arundinaceum, Panicum antidotale, Thysanolaena latifolia*, and *Neyraudia reynaudiana* were selected to verify their potential as a substrate to produce hydrolytic enzymes and to evaluate them as second generation energy biomass. Here, cellulase and hemi-cellulase producing three endophytic bacteria (*Burkholderia cepacia* BPS-GB3, *Alcaligenes faecalis* BPS-GB5 and *Enterobacter hormaechei* BPS-GB8) recovered from *N. reynaudiana* and *S. arundinaceum* were selected to develop a triculture (CC3) consortium. During 12 days of submerged cultivation, a 55–70% loss in dry weight was observed and the maximum activity of β-glucosidase (5.36–12.34 IU) and Xylanase (4.33 to 10.91 IU) were observed on 2nd and 6th day respectively, whereas FPase (0.26 to 0.53 IU) and CMCase (2.31 to 4.65 IU) showed maximum activity on 4th day. Around 15–30% more enzyme activity was produced in CC3 as compared to monoculture (CC1) and coculture (CC2) treatments, suggested synergetic interaction among the selected three bacterial strains. Further, the biomass was assessed using Fourier-transform infrared spectroscopy (FTIR) and Scanning electron microscopy (SEM). The FTIR analysis provides important insights into the reduction of cellulose and hemicellulose moieties in CC3 treated biomass and SEM studies shed light into the disruption of surface structure leading to access of cellulose or hemicelluloses microtubules. The hydrolytic potential of the CC3 system was further enhanced due to reduction in lignin as evidenced by 1–4% lignin reduction in biomass compositional analysis. Additionally, laccase gene was detected from *A. faecalis* and *E. hormaechei* which further shows the laccase production potential of the isolates. To our knowledge, first time we develop an effective endophytic endogenous bacterial triculture system having potential for the production of extracellular enzymes utilizing *S. arundinaceum* and *N. reynaudiana* as lignocellulosic feedstock.

## Introduction

The renewable carbon sources (e.g., lignocellulosic biomass, wastewater) have became the subject of intensive research as an alternative energy source for the generation of clean and sustainable energy. Their use for the production of biofuels in a biorefinary relies solemnly on the enzymatic hydrolysis of pre-treated biomass to produce reducing sugars. A set of enzymes required for synergistic lignocellulosic biomass conversion includes cellobiohydrolases, endoglucanases, beta-glucosidases along with newly described oxidative enzymes (Horn et al., 2012). The major bottlenecks are the cost of enzymes for enzymatic hydrolysis and the fermentation of pentoses from hemicelluloses hydrolysis (Camassola and Dillon, 2009; Koppram et al., 2012). The potential solution is the on-site production of these enzymes in order to minimize the expenses of enzymes addition externally (Kazi et al., 2010). One way by which it can be achieved is the development of microbial coculture systems of compatible fungal or bacterial strains (Kolasa et al., 2014).

The perennial grasses are considered as a rich source of lignocellulosic biomass, making it a second generation alternative energy source and can diminish the use of fossil fuels. Traditional forage grasses, e.g., *Saccharum arundinaceum, Panicum antidotale, Thysanolaena latifolia*, and *Neyraudia reynaudiana*, can also have the potential to produce energy. In addition, all of them are abundantly available on the road sides and forests, and can be harvested minimum three times a year, which makes them a suitable plant for the production of energy (Bor, 1960; Nair and Sekharan, 2009).

Role of cellulases and xylanases are crucial in the degradation of lignocellulosic materials and often are required in large quantities (Wongwilaiwalina et al., 2010; Zhou et al., 2010; Scholl et al., 2015). The cellulase complex consists of endoglucanases, exoglucanases and glucosidases, which plays an important role in biomass hydrolysis to simple sugars like glucose (Ogeda and Petri, 2010). In this context, a highly promising source of activity is the cellulolytic enzymes (Medie et al., 2012). The enzymes of xylanolytic complex are responsible for the conversion of main carbohydrate xylan found in hemicellulose to xylose (Kuhad et al., 1997). The cellulases and xylanases are mainly used in textile industry for denim fading and in pulp and paper, baking along with animal feed industries respectively. Therefore, the use of microorganisms that produce hydrolytic enzymes such as cellulases and xylanases, and low cost abundantly available substrates, such as perennial grasses are probable candidates for making second-generation biofuel.

Endophytic microorganisms are recognized as a new source of genes, enzymes (cellulases and xylanases) and other secondary metabolites to assist their adaption and survival within higher plants (Cho et al., 2008; Xiong et al., 2013; Castro et al., 2014). It is showed that hydrolytic enzymes produced by endophytes are involved in the initial infection process of the host (Hallmann et al., 1997). However, they also produce these extracellular hydrolyases in order to establish a resistant mechanism against plant invasion, thus can have beneficial effect on host plant (Tan and Zou, 2001). In relation to cellulases and xylanases *Alcaligenes faecalis* is remarkable for degradation of hemicellulose and cellulose by 73.5% and 67.3% respectively in a coculture system (Yang et al., 2011). Earlier β-glucosidase from *A. faecalis* was purified and has a capability to hydrolyse a wide variety of different chemical types (Day and Withers, 1986). Similarly, a lipase is been isolated and purified from *Burkholderia cepacia* RQ3 with broad solvent ability with an excellent enantio selective transesterification (Xie et al., 2015). *Enterobacter hormaechei* along with *Escherichia fergusonii* was the predominant species for hydrogen production from paperboard mill wastewater (Farghaly et al., 2015).

Among the endophytic microorganism few endophytic fungi showed lignocellulosic biomass degrading efficiency (Dai et al., 2010; Purahong and Hyde, 2011). However, very few reports are available on endophytic bacteria having potential to degrade lignocellulose resources (Ma et al., 2016). Recently, Xiong et al. (2013) had demonstrated that an endophytic bacteria *Pantoea ananatis* Sd-1 isolated from rice seeds with strong lignocellulosic biomass degradation ability to degrade rice straw and lignin. Due to the rapid growth, environmental adaptability and biochemical versatility of bacteria (Archana and Mahadevana, 2002), developing an endophytic bacterial system may provide a variety of advantages in lignocellulosic biomass degradation as compared to fungi.

In this context, this study evaluated the production of hydrolytic enzymes by the endophytic bacteria obtained from selected two perennial grasses *S. arundinaceum* and *N. reynaudiana* in submerged cultivation. The obtained bacterial isolates were screened and quantified for the production of cellulases and xylanases. The potential bacterial isolates were targeted as monoculture, co-culture, and triculture systems to identify the best genotypes with potential to degrade the biomass by the production of hydrolytic enzymes for the generation of second-generation biofuels. Our findings suggested a tri-culture system (CC3) with high efficiency to degrade lignocellulosic biomass through submerged fermentation.

## Materials and methods

### Collection of raw biomass materials

The above ground parts of the four perennial grass samples *S. arundinaceum* (BPS-G101), *P. antidotale* (BPS-G102), *T. latifolia* (BPS-G104), *N. reynaudiana* (BPS-G109) were collected from Murlen National Park (23°37′01″N and 93°18′00″E) using local forage chopper machine. The leaves and stems were dried separately at 55°C in a hot air oven and were chopped into smaller pieces by a chopper, followed by grounding into smaller particles using hammer mill (Barbender Rotary Mill, Type: 880805, Germany) and finally particles of size ranging between 0.5 to 5.00 mm was obtained by using 20 mesh sieve (Deswal et al., 2011; Menegol et al., 2014).

### Isolation and qualitative screening for the production of hydrolytic enzymes

Tested bacterial isolates were obtained from endosphere tissues of *S. arundinaceum* (BPS-G101) and *N. reynaudiana* (BPS-G-109) by using the method of Sturz et al. (1998). The grass tissues (leaves and stems) were collected and brought into the laboratory and washed thoroughly in running tap water to remove all dust particles. Tissues were rinsed for 30 s in 95% ethanol solution followed by a rinse with sodium hypochlorite solution (2% available Cl^−^) for 5 min. Finally three washes were given with the sterilized double distilled water and tissues were dried under laminar airflow. To check the efficiency of sterilization process and to verify that no biological contamination was there on the surface of the tissues, sterilized pieces of tissues were pressed onto the tryptic soy agar (TSA) medium and aliquots of water from the last wash solution was spreaded on TSA plates and examined for the contaminants. Tween 20 (1 mL L-1) was used as surface-tension depressant in all the hypochlorite and rinsing solutions. The dried treated tissues were placed on Luria Bertani (LB) medium and observed for bacterial growth. The isolates emerging from the tissues were carefully selected and were purified by repeated streaking. All the isolates were screened for the production of cellulase and xylanases by using Congo red assay as described by Teather and Wood (1982) and laccase by using ABTS and syringaldazine as substrate (Yaver et al., 1996; Wang et al., 2010).

### Identification of endophytic bacteria

The potential isolates were identified by 16S rRNA gene amplification using universal 16S rRNA primers PA: 5′-AGAGTTTGATCCTGGCTCA-3′ PH: 5′- ACGGCTACCTTG TTACGACT-3′) (Qin et al., 2009). Amplification was done by using Veriti thermal cycler (Applied Biosystems, Singapore) in a total volume of 25 μl containing 1x PCR buffer with 15 mM MgCl_2_ (Fermentas, Canada Inc.), 200 μM of each dNTP, 0.2 μM of each primer, 1U Taq polymerase (Fermentas, Canada Inc.) and 50 ng of genomic DNA. The thermal cycler was programmed as follows: an initial denaturation at 94°C for 5 min, followed by 30 cycles of denaturation at 94°C for 1 min, annealing at 57°C for 1 min and extension at 72°C for 1.2 min with a final extension at 72°C for 10 min. The amplified PCR products were visualized by electrophoresis in 1.5% agarose gels and documented using a Bio-rad Gel Doc XR^+^ system (Hercules, CA, USA). The PCR products were purified using Purlink PCR Purification Kit (In-vitrogen), and were sequenced commercially at SciGenom Labs Pvt. Ltd, India.

### Compatibility test for selected organisms

The drop plate-direct count method was employed to verify the bacterial density of the single, co- and tri-culture systems of the selected isolates for their compatibility to survive in synergistic manner. Selected endophytic bacterial isolates were grown in a modified CMCase and xylanase induction broth media (BPS-CX broth containing Peptone 1%; Yeast Extract 0.25%; K_2_HPO_4_ 0.5%; NaCl 0.5%; supplemented with 0.1% CMC, and 0.5% xylan) for 12 days and were incubated at 28°C with continuous shaking at 150 rpm. One milliliter aliquot was taken out from the flasks on 1st, 2nd, 4th, 6th, 8th, 10th, and 12th days and was subsequently diluted to obtain countable colonies (30–300 colonies) on BPS-CX agar media plates. The plates were incubated for 24 h at 28°C before being counted. All experiments were performed in triplicate and the cell densities were expressed in log (CFU/ml) (Maki et al., 2014).

### Biomass pretreatment and characterization

The biomass samples were pretreated by steam explosion as prescribed by Scholl et al. (2015) with minor modifications. The biomass was washed with distilled water, filtered through nylon cloth and centrifuged at 600 rpm for 1 h to remove excess water. The filtrate was air dried in a hot air oven at 50°C for 12 h and was used for further characterization.

#### Determination of the dry weight of the selected grasses

One gram of the selected biomass was placed in a pre-weighed clean crucible. The material was then kept in an oven at 105°C ± 3 for minimum 4 h to remove the moisture content. After that, the material was cooled in a desiccator, weighed, and the dry weight was recorded relative to the dry mass of the original material.

#### Determination of the ash content in the biomass

The ash determination was performed, according to the methodology of the National Renewable Energy Laboratory (NREL–TP–510–42621), via gravimetry using 300 mg of grass that was calcined in the muffle furnace at 575 ± 25°C for 2 h.

#### Extractables

The Extractables were determined via gravimetry according to the methodology of the National Renewable Energy Laboratory (NREL–TP–510–42619).

#### Determination of the total nitrogen and protein content

The total protein and nitrogen content was estimated commercially at the cashew Export Promotion Council (CEPC), of India, Kerala by using the method from AOAC (2012).

#### Chemical analysis of the cellulosic, hemicellulosic, and lignin substrates

The chemical composition of the raw and the pretreated samples were determined according to the methodology of the National Renewable Energy Laboratory (NREL–TP–510–42618).

### Development of Mono, Co, and Tri-culture systems

The selected bacterial isolates based on their hydrolytic zones in CMCase and xylanase plate assays were pre-grown in 50 ml of BPS-CX induction broth at 30°C, 150 rpm for 4 days. Based on the hydrolytic enzyme production ability and compatibility of the selected isolates, three systems (CC1, CC2, and CC3) were developed to understand their efficacy of biomass degradation (Table 1).

**Table 1:** **The mono, co, and tri-culture systems (*Burkholderia cepacia* BPS-GB3, *Alcaligenes faecalis* BPS-GB5, and *Enterobacter hormaechei* BPS-GB8**.

**Sl. No**.	**Microbial consortium**	**Microbial combinations**
1.	CC1a	BPS-GB3
2.	CC1b	BPS-GB5
3.	CC1c	BPS-GB8
4.	CC2d	BPS-GB3 + BPS-GB5
5.	CC2e	BPS-GB3 + BPS-GB8
6.	CC2f	BPS-GB5 + BPS-GB8
7.	CC3g	BPS-GB3 + BPS-GB5 + BPS-GB8

### Biomass utilization assay

The pretreated grass biomass (PGB) samples were subjected to submerged fermentation (SmF) for 12 days in all three systems. One ml of bacterial suspension was added in 350 ml of BPS-YM Media (1% Peptone and 0.5% Yeast extract in Phosphate Buffer pH 7) supplemented with 5% of the PGB in all three systems. The resulting suspension was incubated at 30°C with continuous agitation at 150 rpm. Aliquots were taken out on alternative days till 12 days to carry out the enzymatic assays. On cultivation the supernatant was filtered out through a sterile nylon cloth and was centrifuged at 6000 rpm at 4°C for 15min. The supernatant was subjected to further crude enzyme preparations. The remaining biomass was dried at 55°C for 48 h and the Relative Dry Weight (RDW) was calculated with the untreated PGB serving as the control (Haruta et al., 2002).

### Crude enzyme preparations and enzyme assays

The supernatant obtained after SmF treatment by all 3 systems, were further centrifuged at 10,000 rpm for 15 min at 4°C. This cell free supernatant was used to quantify the FPase, CMCase, β-glucosidase, and xylanase activities. To determine the cellulase levels, a filter paper activity (FPA) assay was performed according to Ghose (1987), with modifications from Camassola and Dillon (2012). The FPA units were defined as the amount of enzyme capable of releasing 1 μmol of reducing sugar per min per ml. Endoglucanase activity was detected by following the method of Ghose (1987). One unit of endoglucanase activity was defined as the amount of enzyme capable of releasing 1 μmol of reducing sugar per min per ml. For the β-glucosidase activity, p-nitrophenyl-D-glucopyranoside (p-NPG) was used as the substrate. One unit of β-glucosidase activity (using the substrate pNPG) was defined as the amount of enzyme required to hydrolyze 1 μmol of p-NPG per min to release p-nitrophenol (p-NP) per ml. To determine the xylanase activity following the method of Bailey et al. (1992), using 1% xylan (w/v) as the substrate. One unit of xylanase activity was defined as the amount of enzyme capable of releasing 1 μmol of xylose per min per ml. All enzymes activities were represented in IU. One unit (IU) of enzyme activity was defined as the amount of enzyme that released 1 μmol of reducing sugar in a minute reaction. All samples were analyzed in triplicate and mean values were calculated.

### Fourier transformed infrared spectroscopy (FTIR)

FTIR spectra of pretreated and CC3 treated biomass were obtained at room temperature, using a JASCO FT/IR-6800 Spectrometer (Jasco, Japan) equipped with an Attenuated Total Reflectance unit. Spectral data between 650 and 4000 cm^−1^ were collected averaging 64 scans at a resolution of 4 cm^−1^. Principal components analysis (PCA) was performed with KnowItAll ID Expert (Biorad, USA).

### Morphology of biomass after decomposition using SEM

Scanning electron microscopy (SEM) was used to observe the cell surface morphology of the pretreated and CC3 treated biomass. Milled samples were dried before coating with carbon in a Balzers SCD 050 sputter coater (BAL-TEC AG, Balzers, Liechtenstein). Samples were viewed using a scanning electron microscope model Quanta 250-FEG (FEI, Czech Republic). Milled samples were dried and applied on the double side carbon pasted on an aluminum stub. The aluminum stub containing the sample was placed in the sample chamber of ESEM (Environmental Scanning Electron Microscope). After attaining high vacuum, various parameter like electron beam, intensity, spot size, voltage, emission current were adjusted and the images were captured. A large number of images were obtained from different areas of the samples (at least 20 images per sample) to confirm the reproducibility of results.

### PCR based detection of laccase gene

The laccase gene was amplified from the laccase producing isolates using *cotA* gene specific primers, CotA F: 5′ TTAG GATCCATGAACCTAGAAAAATTTGTTGACG3′ and cotA R: 5′ CCCAAGCTTCTAAATAATATCCATCGGCCGCAT3′ primers (Su et al., 2013). The PCR reaction mixture (15 μl) contained, 7.5 μl of 2X Emerald Amp GT PCR Master mix (Takara, Japan); 1.0 μl of each primer (10 pmol) and bacterial DNA (100 ng). The PCR was carried out with initial denaturation of 94°C for 5 min followed by 35 cycles of 94°C for 1 min, 57°C for 1 min and 72°C for 1 min and ending with a 10 min final extension at 72°C. PCR reactions were run on a 1.5% agarose gel in 1X TAE.

## Results

### Screening and isolation of endophytic bacteria for hydrolytic enzyme production

In total 15 bacterial isolates were recovered from the endosphere tissues of *S. arundinaceum* (BPS-G101) and *N. reynaudiana* (BPS-G109). All the isolates were purified by repeated streaking and were screened for the production of cellulase and xylanase on CMC and Xylan (Oat Spelt xylan) containing agar plates (Table 2). Among the isolates two isolates (BPS-GB3 and BPS-GB5) were positive for both CMCase and xylanase activity, while isolate BPS-GB8 showed only CMCase activity, by producing a clearing zones on respective medium by using Congo Red Assay.

**Table 2:** **Qualitative screening for Cellulase and Xylanase enzymes among the endophytic isolates as indicated by hydrolysis Zone (+: 0.2–0.5 mm, ++: 0.6–1.0 mm, +++: 1.1–1.2 mm)**.

**Endophytic bacteria**	**Hydrolytic zone in mm**	
	**CMCase**	**Xylanase**
BPS-GB1	−	−
BPS-GB2	−	−
BPS-GB3	+++	+
BPS-GB4	−	−
BPS-GB5	++	−
BPS-GB6	−	−
BPS-GB7	+	−
BPS-GB8	+	+++
BPS-GB9	−	−
BPS-GB10	−	−
BPS-GB11	−	+
BPS-GB12	−	−
BPS-GB13	−	−
BPS-GB14	−	−
BPS-GB15	+	−

### Lignolytic enzyme activity in endophytic bacteria

**Figure 1:**
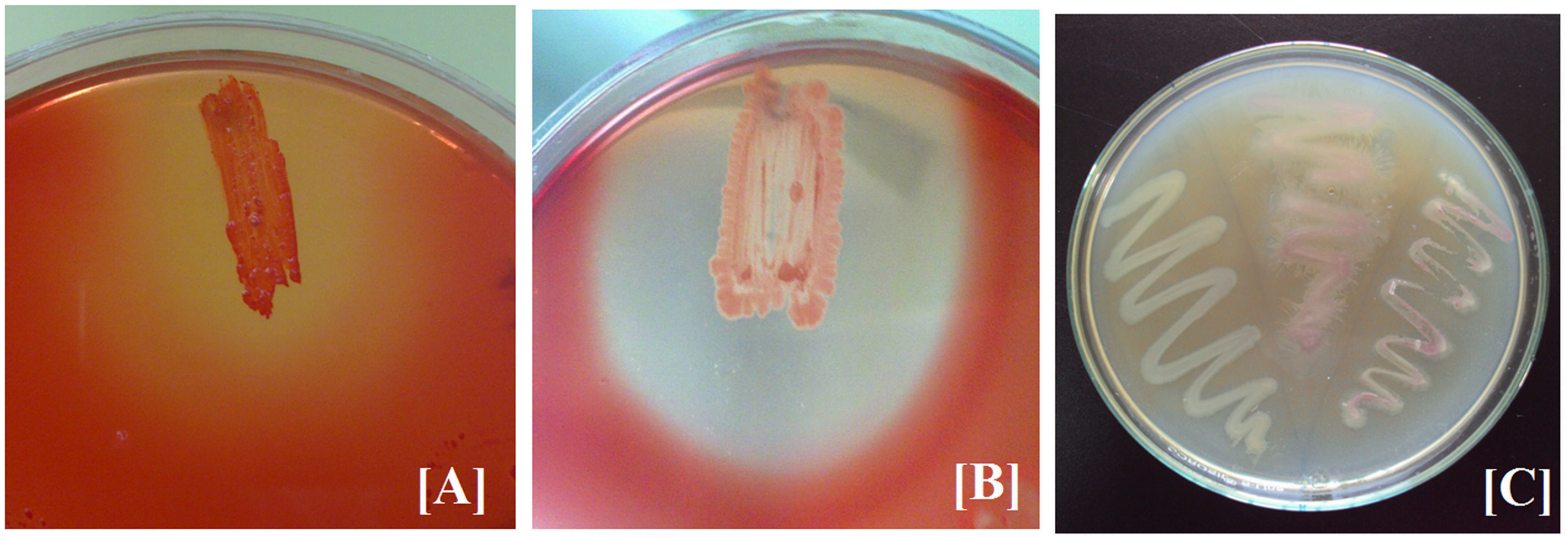
**Screening of Extracellular Enzyme production: (A) cellulase, (B) xylanase, and (C) Cell bound laccase of selected endophytic isolates**.

**Figure 2:**
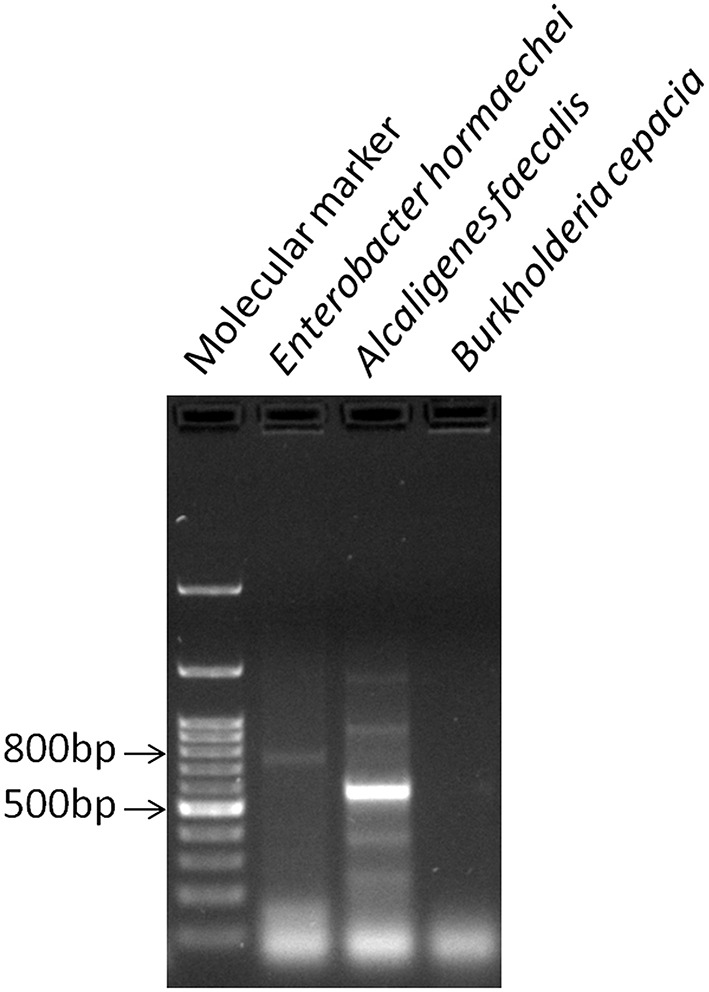
**PCR based detection of laccase gene (cotA) in BPS-GB8 (*E. hormaechi*) in BPS-GB5 (*A. faecalis*)**.

The three bacterial cultures (BPS-GB3, BPS-GB5, and BPS-GB8) which were positive for cellulase and xylanase were screened for bacterial laccase activity using ABTS and syringaldazine as substrate. Absence of green zone in ABTS containing plates during bacterial growth suggested lack of laccase activity. But in case of syringaldazine applied onto bacterial colonies, two of the cultures (BPS-GB5 and BPS-GB8) oxidized the syringaldazine due to laccase like activity in the cells (Figure 1). This was confirmed by PCR screening for the presence of laccase gene in the bacterial cultures using *cotA* gene specific primers. The resultant amplified product of about 750 bp in BPS-GB8 and 600 bp in BPS-GB5 suggest that laccase activity is present in these cultures. But no amplification was seen in BPS-GB3 (Figure 2).

### Identification of potential endophytic bacteria

**Figure 3:**
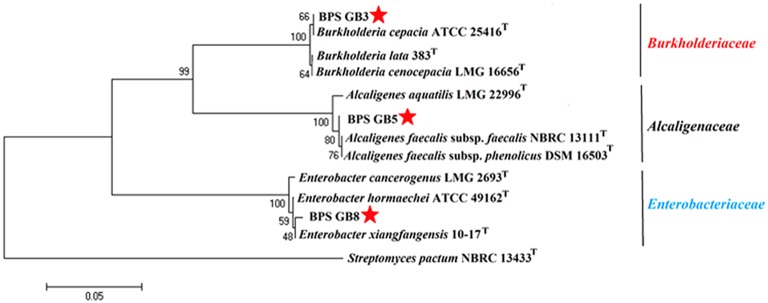
**Neighbour-joining phylogenetic tree based on 16S rRNA gene sequences of selected endophytic bacteria showing the relationship between closest type strain sequences**. Number at branches indicate bootstraps value (>50%) from 1000 replicates.

The potential isolates were identified by using microscopic, biochemical and molecular genetic analysis. All three selected bacterial isolates were gram negative and were positive for catalase, cellobiose, and nitrate reduction (Table 3). The partial 16S rRNA gene sequence of the three isolates has been analyzed by using EzTaxon database and a phylogenetic tree was constructed by using Mega 5.5. Isolate BPS-GB3, BPS-GB5, and BPS-GB8 showed a high identity with *B. cepacia, A. faecalis*, and *Entrobacter hormaechei*, respectively (Figure 3). 16S rRNA gene sequences were deposited in NCBI-GenBank and the accession numbers were obtained as KU158235, KU158236, and KU158237.

**Table 3:** **Morphological and biochemical test for characterization of the potential hydrolytic enzyme producing endophytic bacterial isolates**.

**Morphological and biochemical test**	***Burkholderia cepacia* (BPS-GB3)**	***Alcaligenes faecalis* (BPS-GB5)**	***Enterobacter hormaechei* (BPS-GB8)**
**Shape**	**Rods, Gram-negative**	**Rods, Gram-negative**	**Rods, Gram-negative**
VP test	−	−	+
Glucose	+	−	+
Fructose	+	−	+
Sucrose	+	−	+
Xylose	+	−	+
Cellobiose	+	−	+
Oxidase	−	+	−
Citrate utilization	+	+	+
Nitrate reduction	−	−	−
Urease test	−	−	+
Catalase	+	+	+
H_2_S production	−	−	−

### Compositional analysis of the selected grass biomass

The pretreated biomass was analyzed for % of extractives, protein, cellulose, hemicellulose, pectin, lignin, ash, and moisture contents (Table 4). Cellulose content among the selected biomass had shown large variations ranging from 36.51 to 48.74%, which further increased in the pretreated biomass to 49.31 to 53.70%, suggest that the biomass is amenable for deconstruction and release of macromolecules (Tables S1, S2). Comparing the composition of various biomasses used for production of second generation biofuels, it appears that the biomasses used in the present study has great potential due to high concentration of cellulose, hemicellulose and low lignin content (Table 4), which seems to be a good source for obtaining reducing sugars like glucose.

**Table 4:** **Comparison between the composition of holocellulose and lignin contents in the raw perennial grasses studied as compared to other energy crops reported**.

**Biomass**	**Compositional Analysis**			**References**
	**Cellulose (%)**	**Hemicellulose (%)**	**Lignin (%)**	
*S. arundinaceum* (BPS-G101)	36.51 ± 0.16	25.35 ± 0.44	8.19 ± 0.17	This work
*P. antidotale* (BPS-G102)	43.46 ± 0.12	20.53 ± 0.56	11.02 ± 0.15	This work
*T.latifolia* (BPS-G104)	48.74 ± 0.16	17.74 ± 0.65	8.70 ± 0.54	This work
*N. reynaudiana* (BPS-G109)	38.69 ± 0.23	22.24 ± 0.77	11.64 ± 0.49	This work
*Panicum purpureum*	45.97 ± 3.10	27.03 ± 1.02	22.80 ± 1.26	Lima et al., 2014
*Panicum virgatum*	35.7 ± 3.20	27.6 ± 2.02	25.9 ± 1.16	Galletti et al., 2015
*Panicum maximum*	39.87 ± 1.97	26.62 ± 1.46	25.36 ± 1.06	Lima et al., 2014
*Brachiaria brizantha*	43.48 ± 1.84	23.23 ± 3.16	23.09 ± 0.73	Lima et al., 2014
*Arundo donax*	41.6 ± 1.04	23.60 ± 0.87	24.6 ± 1.86	Galletti et al., 2015
*Miscanthus floridulus*	32.71 ± 3.55	34.86 ± 2.91	8.90 ± 1.98	Qin et al., 2012
*Miscanthus sinensis*	35.06 ± 4.33	34.82 ± 3.26	9.51 ± 1.82	Qin et al., 2012
*Miscanthus lutarioriparius*	42.11 ± 6.19	32.34 ± .43	13.64 ± 2.33	Qin et al., 2012
*Miscanthus sacchariflorus*	38.50 ± 4.24	32.98 ± 4.06	11.22 ± 2.24	Qin et al., 2012
Sugarcane baggase	39.44 ± 1.21	27.45 ± 2.08	27.79 ± 1.39	Lima et al., 2014

### Compatibility test for selected organisms

**Figure 4:**
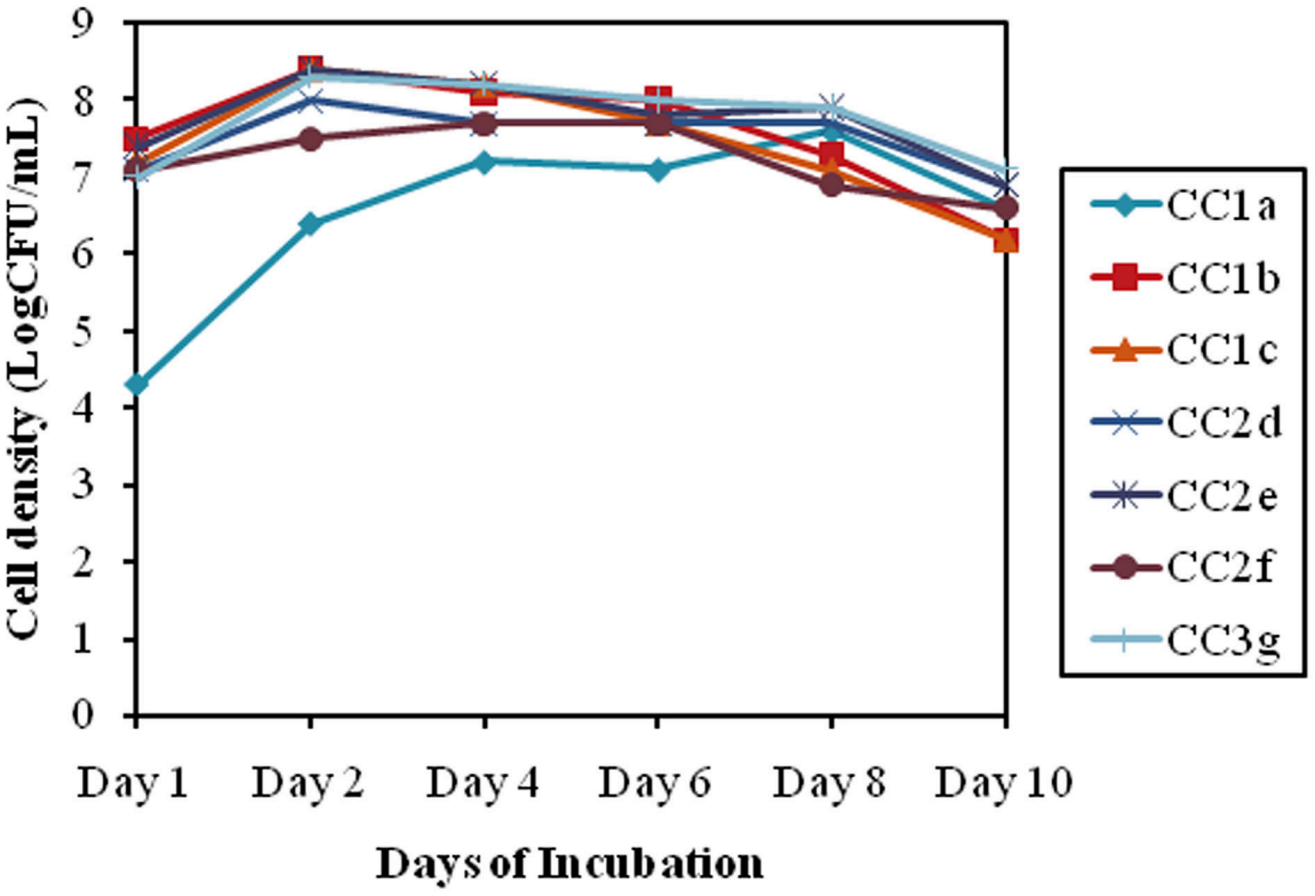
**Bacterial consortium synergistic growth patterns, indicated by the log (CFU/mL) for alternative days of incubation**.

To verify the synergistic effect of the selected isolates, a compatibility test was done which revealed that the bacterial consortium showed a similar growth pattern as comparison to the individual growth pattern. The cell densities for the selected individual isolates showed approximately 7.2 log (CFU/ml) till 3 days of inoculation. Additionally after 3 days all mixes (CC2 and CC3) recorded an approximately similar growth pattern averaging around 8.2 ± 0.2 log (CFU/ml) till 10 days (Figure 4).

### Enzyme production by selected endophytic bacterial isolates

The three potent strains showing hydrolysis zone were further selected for enzyme quantification and were grown in basal induction broth media supplemented with 2% Filter paper pulp, 0.3% of CMC, 0.3% Beechwood xylan, and 0.1% of p-nitrophenyl-D-glucopyranoside (p-NPG) for the estimation of FPase, CMCase, xylanase, and β-glucosidase, respectively. The enzyme production was quantified concurrently on 1st day followed on alternative days from day 2 till day 10. The results revealed that BPS-GB3 produced the maximum CMCase (1.82 IU/mL) and FPase (0.370 IU/ml) in day 2 and day 4 of incubation. BPS-GB5 was the only isolate that expressed β glucosidase activity starting from day 1 to day 4, with the optimum enzyme production denoted at day 2 of incubation with 5.50 IU/mL. While BPS-GB8 exhibited maximum xylanase (6.66 IU/mL) on day 6 of incubation (Table S3).

### Biomass utilization by co-culture systems

Based on the enzyme production capabilities of the three strains, three co-culture systems were prepared and their effectiveness in biomass utilization and degradation was verified on the selected four feedstocks. Among the co-culture systems, CC2e treated feedstock (BPS-G109) showed the highest xylanase (8.45 IU/mL) production, whereas high FPase (0.37 IU/mL) was recorded in BPS-G101. The CC2d system showed significant production of CMCase (0.154 to 1.85 IU/mL) and β-glucosidase (5.5 to 11.33 IU/mL) from day 1 to day 6 in all the four selected feedstocks, with a maximum effect on BPS-G102 and BPS-G109. CC2f system showed the very marginal activities for xylanase and FPase production as compared to other co-culture systems. Unfortunately, none of the tested co-culture system was able to produce all four hydrolytic enzymes under study. Hence, the tri-culture system was developed to look for the possibilities of having more efficient system having enhanced enzymes productions (Table S3).

### Biomass utilization by triculture (CC3) system

Based on the results obtained in co-cultures systems, a tri culture system was designed to look for enhanced enzyme production. The maximum β-glucosidase activity was observed at day 2 of incubation in all substrates treated with CC3 culture system. Maximum β-glucosidase production was observed in both BPS-G101 and 109 substrates with 12.34 and 12.22 IU/mL, respectively. β-glucosidase activity declined from day 6 in all enzyme extracts. The highest CMCase activity was recorded among BPS-G101 and BPS-G109 on day 2 (0.23 IU/ml) and day 4 (2.3 IU/ml) respectively. Among the selected biomasses, BPS-G101 and BPS-G109 showed relatively stable CMCase activity from day 2 to day 6 of incubation however, BPS-G102 showed highest CMCase (4.650 IU/ml) activity on 2nd day of incubation, which drastically diminished after day 4 of incubation.

**Figure 5:**
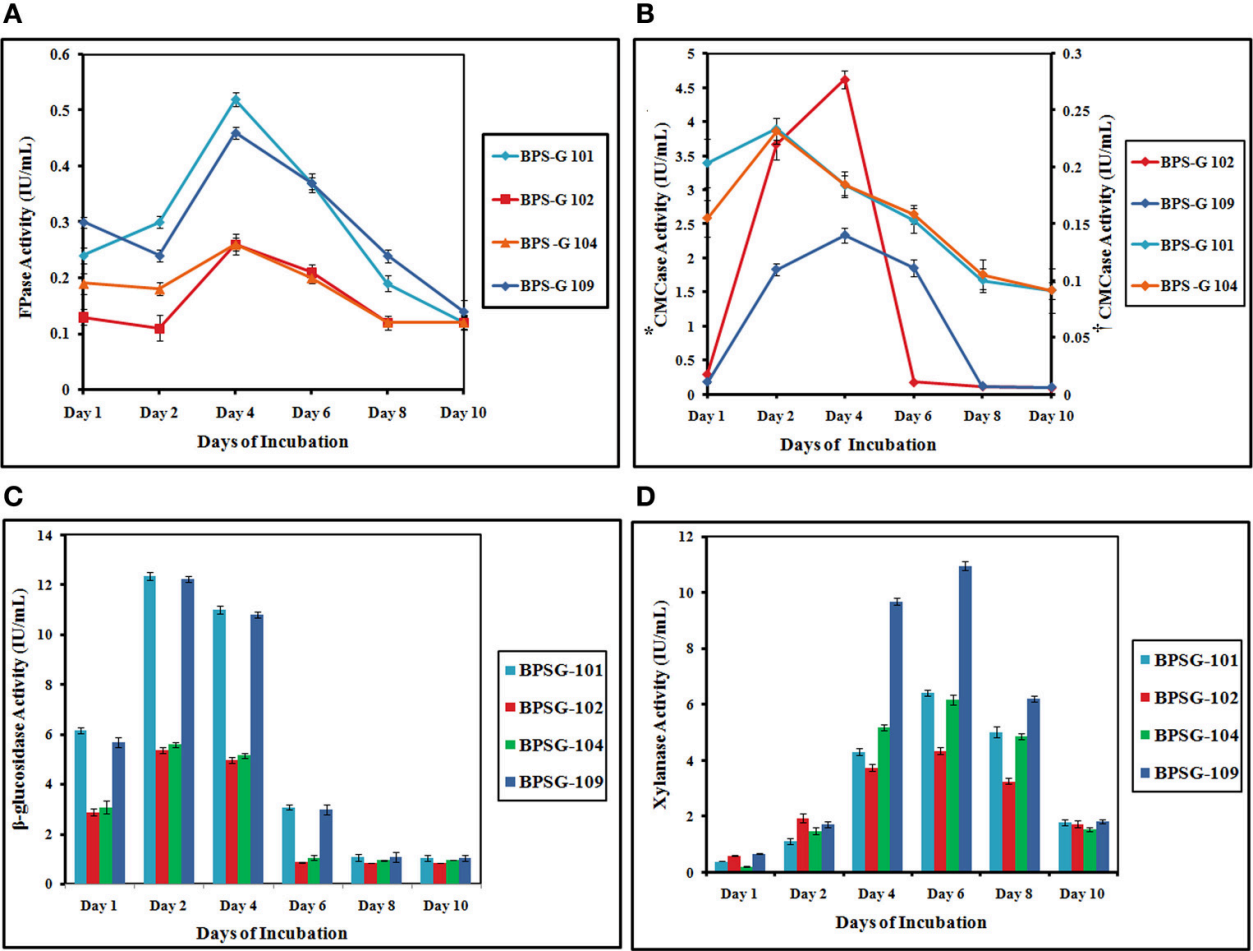
The FPase (A), CMCase (B^*,†^), β-glucosidase (C) and Xylanase (D) activities of the CC3 system on pretreated 5% (w/v) biomass studied [BPS-G101, G102, G104 and G109] at different time intervals. ^*^Primary y axis- CMCase Activity of CC3 treated BPS-G102 and BPS-G109; ^†^secondary y axis- CMCase Activity of CC3 treated BPS-G101 and BPS-G104.

Similarly, FPase activity was found to be highest in CC3 treated BPS-G101 (0.528 IU/mL) and BPS-G109 (0.46 IU/mL) on the 4th day of incubation. However, xylanase activity was optimum on day 6 in all CC3 treated biomasses. BPS-G101 (6.41 IU/mL) and BPS-G109 (10.91 IU/mL) recorded the significant xylanase activity as compared BPS-G102 (4.33 IU/ml) and BPS-G 104 (6.13 IU/ml). In all the selected biomasses, CC3 system showed fairly stable xylanase activity from day 4 till day 8, with the maximum activity observed on day 6 of incubation. Thus the results suggested that feedstocks BPS-G101 and BPS-G109 are efficient substrate on which the CC3 system can grow synergistically and produce cellulose and hemicelluloses hydrolyzing enzymes especially β- glucosidases and xylanases in optimal amounts with CMCase and FPase production as well (Table S3 and Figure 5).

### Biomass relative dry weight loss

**Figure 6:**
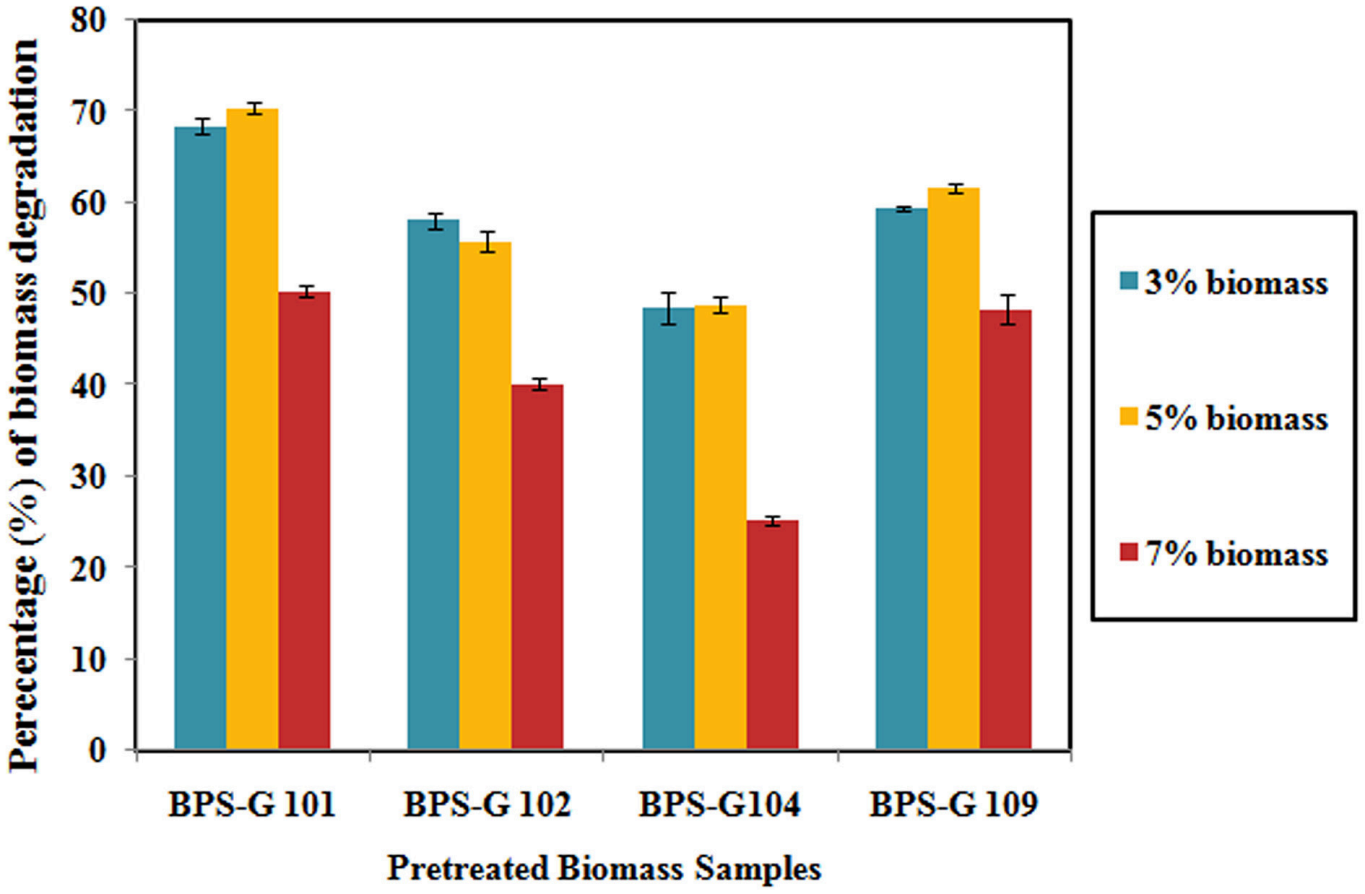
**Decomposition of the selected biomass (BPS-G101, G102, G104, and G109) by CC3 system at varying concentration of 3, 5, and 7% (w/v) expressed as the dry weight percentage**.

About 48 to 70% loss in dry weight was observed in the feedstocks used in the present study, among them BPS-G101 (70%) and BPS-G109 (61%) found the maximum relative dry weight loss. Among the tested different concentrations (3, 5, and 7%) of feedstocks, 5% feed of biomass was found to be more efficient in CC3 culture treatments as calculated by the relative dry weight loss after 10 days of incubation as compared to control (Figure 6).

### Scanning electron microscopy (SEM) analysis

**Figure 7:**
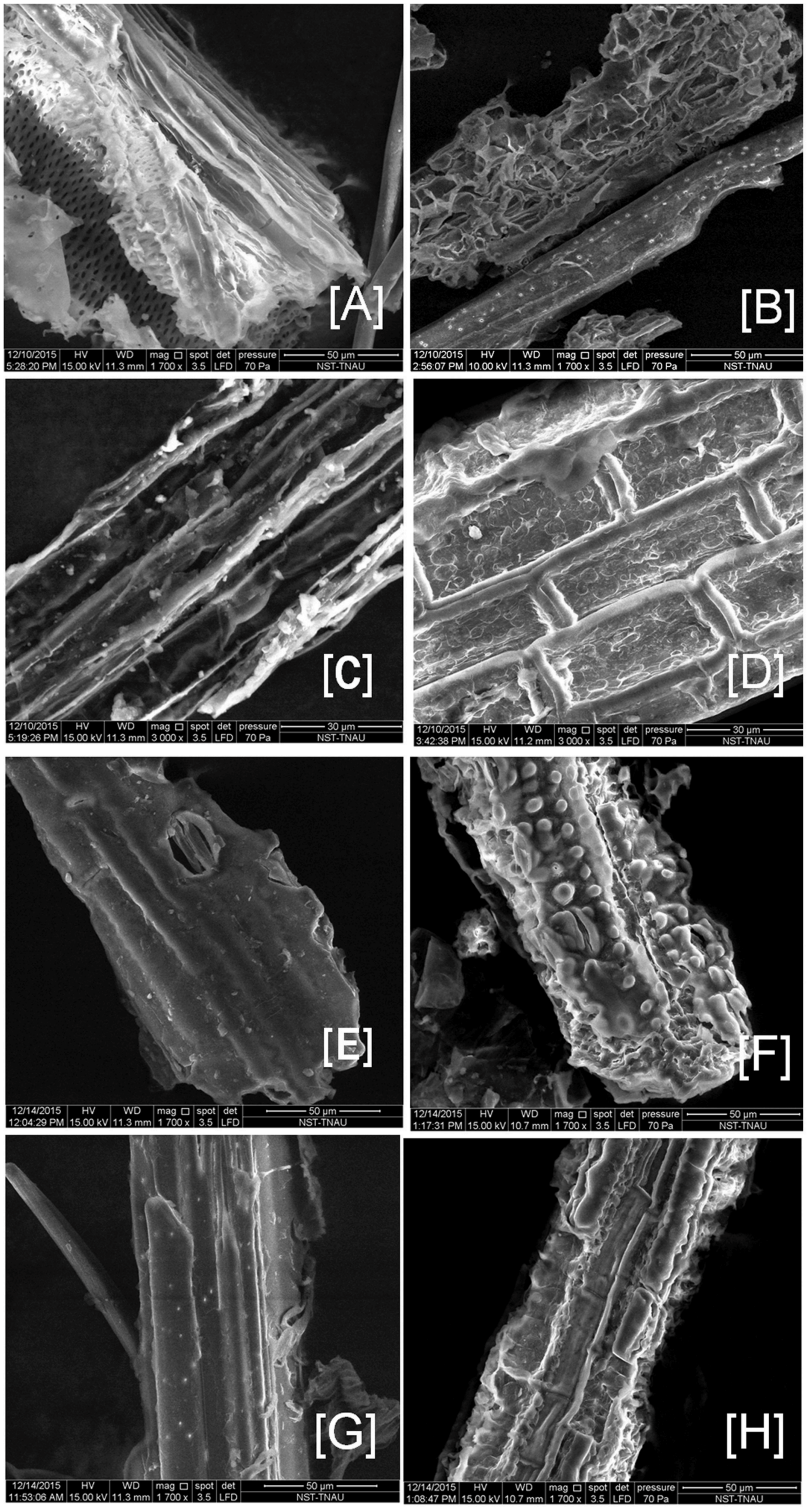
**Scanning electron microcopy image of the CC3 treated biomass BPS-G101 (B,D) and BPS-G109 (F,H) and compared to their respective pretreated biomass as control (A,C,E,G)**.

Based on the degradation potential of tri-culture system (CC3), the two best feedstocks (BPS-GB-101 and 109) were selected for SEM analysis to look changes in their morphology after 10 days of incubation and was compared with control. Pretreated feedstock was served as control. Figure 7 clearly showed disintegration of cell layers and formation of channels and gaps in the CC3 treated feedstocks as compared to the control. This clearly indicates the potential of the selected system in the degradation of feedstocks (Figure 7).

### Fourier-transform infrared spectroscopy (FTIR) analysis

**Figure 8:**
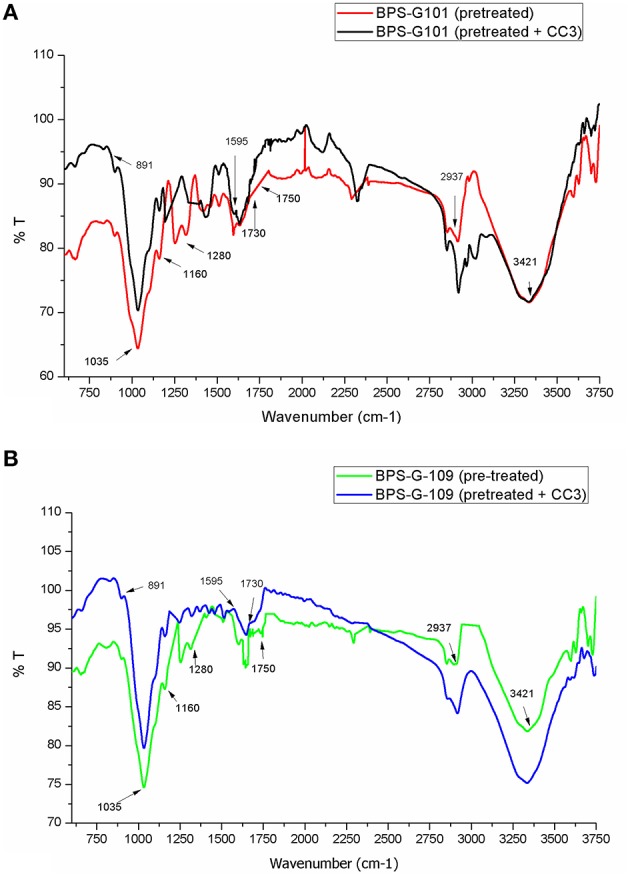
**FT-IR spectroscopy of the pretreated biomass BPS-G101 (A) and BPS-G109 (B) and their CC3 treatments**.

FTIR spectral analysis was carried out for pretreated and CC3 treated feedstocks (BPS-G101 and BPS-G109) to understand the compositional and structural changes occurred during feedstock digestibility. The samples were analyzed in the spectral region of 650 to 4000 cm^−1^. Small differences in the intensity of the peak at 1160 cm^−1^ related to the C-O-C stretching in cellulose and hemicellulose were observed, while a slightly significant decrease of the peak at 1035 cm^−1^ was detected indicating the C-O stretching of cellulose in both CC3 treated BPS-G101 and G109. There was almost complete absence of a peak at 1280 cm^−1^ in the CC3 treated samples which denoted the C-H bending for crystalline cellulose. Characteristic assignment of hemicellulose at 1738/1730 cm^−1^ (C = O conjugates in xylans) was only observed in pre-treated biomass samples of BPS-G109 and 101.The FTIR analysis indicates a reduction of cellulose and hemicellulose moieties which are in concordance with the hydrolysis enzymes produced during CC3 treatments. At 891 cm^−1^ the characteristic of the β-glycosidic bond (C-H glycosidic deformation and O-H bending takes place between the anhydroglucose units of the cellulose units of the fibers) have disappeared in the CC3 treatments for both the biomass samples (Figure 8). The disappearance of peaks at 1595 cm^−1^ (aromatic ring vibration = O stretching) and 2937 cm^−1^ (C-H stretching) corresponding to lignin in CC3 suggest the reduction in lignin compared to pre-treated biomass sample and was evidenced by 1–4% lignin reduction by in biomass compositional analysis (Table 5).

**Table 5:** **Lignin content analysis of the Raw, Pretreated and CC3 treated biomass samples (BPS-G101 and BPS-G109) to verify the cell bound laccase activity effects in the CC3 system**.

**Sample name**	**Lignin (%)**		
	**Raw**	**Pretreated**	**Pretreated + CC3**
BPS-G101	8.19 ± 0.17	7.85 ± 0.82	6.74 ± 0.45
BPS-G109	11.64 ± 0.49	10.49 ± 0.95	6.48 ± 0.44

Data presented are mean ± SE from three replicates.

Means are significantly different from control at P = 0.05.

## Discussion

Alternative sources for bioenergy which can be grown sustainably and processed cost effectively are of prime importance in this era of ever diminishing renewable energy resources. The existing bioenergy assets consist of residues from forestry, agriculture, various organic waste and some selected bioenergy crops. One way is to look for a wider range of biomass sources available throughout year, which will solve the problem of operation of biofuel production plants operated seasonally, due to non-availability of sufficient feedstocks. In the longer term, non-fodder lignocellulosic biomass could be the better resources for second generation biofuels (Heather and Somerville, 2012; Gupta et al., 2014). Hence, identifying a biomass that is geophysically popular and endemic will be useful in overcoming the inevitable energy crisis. So, in the present study, the potential of four perennial forage grasses endemic to South Asian countries was demonstrated to use as an efficient raw material for bioconversion studies. Among perennial forage crops, perennial grasses like *Pennisetum purpureum, Phalaris arundinacea*, and *Miscanthus* sp. etc., have shown potential as dedicated bioenergy crops (Gonzalez-Hernandez et al., 2009; Carriquiry et al., 2011). The holocellulose and lignin components play a crucial role in determining the availability of a particular plant in demand as a viable raw material for energy resource studies. A comparison of the selected perennial grasses under study to that of the existing bioenergy crops revealed that all four grasses had least amounts of lignin (Table 3). The holocellulose content was higher as comparable to *Miscanthus* sp, *Panicum* sp, and sugarcane baggase.

Till date, only a few organisms other than white rot fungi had showed the ability to degrade lignocellulosic biomass (Martinez et al., 2009; Xiong et al., 2013). Recent findings suggested that bacterial lignocellulolytic systems may play a more significant role in biomass degradation (Brown and Chang, 2014; Mathews et al., 2015). Development of an efficient system based on endogenous microorganisms would be more specific and effective in biomass degradation. Endophytes reside within the inner tissues of healthy plants without showing any disease symptoms (Strobel and Daisy, 2003). Extracellular enzymes produced by endophytes are the major mean by which they colonize the plant tissues (Hallmann et al., 1997; Kovtunovych et al., 1999). Endophytic bacteria associated with perennial crops are gaining interest for the production of hydrolytic enzymes for biomass conversion potential (Ferreira et al., 2008; Xiong et al., 2013; Castro et al., 2014). Recently, Compant et al. (2005), showed that cell wall degrading enzymes, endogluconase and polygalacturonase are required for immigration of *Burkholderia* sp in *Vitis vinifera*. There are few reports on the application of endophytic bacteria having potential of hydrolytic enzymes production (Lima et al., 2005; Yasinok et al., 2008; Xiong et al., 2013). However, there is no report on endogenous bacteria associated with perennial grasses having potential for biomass degradation.

Many endophytic bacteria have been isolated from various vegetations like medicinal plants (Passari et al., 2015), grasses (Kelemu et al., 2011), plant debris (Hu et al., 2009), and rice (Mano and Morisaki, 2008) were screened for the production of bioactive compounds (Strobel and Long, 1998). Perennial grasses are the most abundant renewable energy source in India. However, there is not much work done to use these grasses as a bioenergy source. This study is mainly focused on the isolation of endogenous bacterial population from the selected grasses and to look for their probability to act as biomass decomposers.

In the present study we identified three endophytic bacterial isolates having the potential to produce hydrolytic enzymes. Isolate *B. cepacia* was found to be positive for the production of both cellulases (CMCase and FPase) and xylanase, which was similarly reported by Al-Gheethi (2015) that *B. cepacia* can synthesize cellulase with varying degrees when grown on CMC agar plates. Similarly, Pessi et al. (2013) also showed that *B. cenocepacia* can produce cellulase, protease and lipase in aerobic and micro-oxic conditions. Though, *Burkholderia* species is well-known for their lipase production capabilities (Rathi et al., 2001), but it has been also reported as phytopathogen (Gonezaleaz et al., 2007), hence there could be a possibility of production of hydrolytic enzymes while infecting the plants. Recently, gene mining studies on *Burkholderia* sp like those associated with *B. cepacia* complex has attained interest (http://www.ncbi.nlm.nih.gov/protein/KWF98908.1). Various cellulase genes have been reported now in the NCBI websites and concurrently in Uniprot (http://www.uniprot.org/uniprot/?query=Burkholderia+cepacia+cellulase&sort=score). These reported findings further prove the capability of the isolate used in the present study for bioconversion. Among the tested endophytic isolates, only *A. faecalis* showed β-glucosidase production (5.50 IU/mL), this is in line with the previous reports on the β-glucosidase production by *A. faecalis* obtained from sugarcane bagasse (Han and Srinivasan, 1969). Recently, *A. faecalis* has been isolated as endophyte from mangrove forest having endo-glucanase activity (Castro et al., 2014). Another selected isolate *Enterobacter hormaechii* showed the production of xylanase. To the best of our knowledge, there are no reports for the production of xylanase from endophytic *E. hormaechii*, though beta-xylosidase (xloA gene) and glucanase (wssD gene) is been reported from *E. hormaechii* ATCC 49162, which further suggests the potential of the isolate as xylanase and glucanase producer (Campos et al., 2014).

Steam explosion method has been reported to be capable of making biomass more accessible for enzymatic action resulting in biodegradation by breaking the lignin-carbohydrate ester bonds and loosened its crystalline structure (Zhao et al., 2012; Scholl et al., 2015). Therefore, in the present study the selected biomasses were pretreated by steam to increase the biodegradable efficiency by using coculture and triculture systems.

Consortium of microbes especially that of bacterial strains capable of surviving together and synergistically produce hydrolytic enzymes have gained immense interest, given their ability to perform more complex tasks and more readily adapt to changes in the environment than mono-culture (Maki et al., 2014). In the present study, an effective system having bioconversion capabilities (for the selected biomass) by producing both holocellulose (cellulose + hemicellulose) degrading enzymes was performed successfully. One major advantage of microbial consortium is the enzymes secreted by various microbes could compensate the absence of another enzyme required for degradation (Maki et al., 2014). This was clearly indicated by the increase in the enzyme activities of the tested CC3 system along with the emergence of all four enzymes whose synergy was absent in mono (CC1) and coculture (CC2) systems. Previously such attempts have been made by several researchers (Wongwilaiwalina et al., 2010; Yang et al., 2011; Maki et al., 2014; Zhang et al., 2014) demonstrated the application of microbial consortium in efficient degradation of lignocellulosic biomass. Zhang et al. (2014) reported the application of a co-culture system of two thermophillic bacterial strains CS-3-2 and CS-4-4 on corn stalk as lignocellulosic substrate belongs to genus *Clostridium* and showed great synergism by increasing glycoside hydrolase secretomes between the two strains. Among the tested systems triculture (CC3) was the most effective treatment of bacterial consortia for biomass degradation. The triculture system was not only capable of producing all four enzyme systems under study, but also the enzymes were expressed in optimal amounts. The enzyme (FPAse, CMCase, β-glucosidase and Xylanase) productions by the CC3 culture system are comparable to that of well-known lignocellulose utilizing fungi like *Penicillium echinulatum* S1M29 acts on Elephant grass (Scholl et al., 2015) and on sugarcane baggase (Schneider et al., 2014). In addition, two of the cultures in the triculture had laccase like activity which helps in the reduction of lignin and thereby loosens the complexity of biomass structure and finally favours sugar recovery.

In continuation, the CC3 system showed enhanced xylanase and CMcase activity as previously reported by Yang et al. (2011), where they have reported activities of 0.21 IU (CMCase) and 3.75 IU (xylanase) by microbial consortia *of Achromobacter xylosoxidians, A. faecalis* and *Fusarium sporotrichioides* actions on switchgrass. Yang et al. (2011) observed a 70% loss in dry weight for 1% of switch grass used for consortia hydrolysis; whereas in the current study a 70% (BPS-G101) and 61% (BPS-G109) loss in dry weight was observed after CC3 treatments for 5% of substrate loading. This further proves the effectiveness of the CC3 system in biomass utilization.

The biodegradation potential of CC3 system showed a 70 and 61% loss in dry weight of BPS-G101 and BPS-G109 respectively, which was in agreement with the findings of Wongwilaiwalina et al. (2010), where they reported a 59 to 77% biomass degradation of sugarcane baggase, rice straw, corn stover and eucalyptus pulp sludge by thermophilic bacterial consortia. The SEM analysis was similar to that of Scholl et al. (2015), where the surface topology of the grass biomass with and without the treatments shed a clearer picture onto the deconstruction of biomass; showing the change of tougher surface to softer particulates, while expansion and loosening of the walls observed resulting in potential enhanced fragmentation.

FTIR spectral data is widely used for chemical analysis of pulp and wood (Ferraz et al., 2000; Chen et al., 2010). The intensities of the holocellulose components with their structural bends and changes in pretreated and CC3 system as showed by FTIR analysis are comparable to findings of Fatah et al. (2014) on oil palm fibers and Lima et al. (2013) on eucalyptus bark digestion by enzymes. The chemical components that build up the biomass are extremely significant in determining their viability as a potent substrate for biofuel production. Variations within biomass lignocellulolytic contents do have a major impact on selecting the enzymes needed to hydrolyze the substrate to simpler sugars as well as their medium composition.

## Author contributions

VL, AP, and VM: Complete the entire experiments and prepared the draft manuscript. JB, SU, RS, and NR: Supported VL to fulfill the experiment and also help in preparing the manuscript. VS, VG, and BS: Analyzed the data and coordinated the study. BS: All the experiment design and checked carefully, written the manuscript and approved the final manuscript.

### Conflict of interest statement

The authors declare that the research was conducted in the absence of any commercial or financial relationships that could be construed as a potential conflict of interest.
